# Effect of Implementing a Free Delivery Service Policy on Women's Utilization of Facility-Based Delivery in Central Ethiopia: An Interrupted Time Series Analysis

**DOI:** 10.1155/2020/8649598

**Published:** 2020-12-12

**Authors:** Ayalneh Demissie, Alemayehu Worku, Yemane Berhane

**Affiliations:** ^1^Department of Public Health, College of Health Sciences, Arsi University, Arsi, Ethiopia; ^2^Department of Biostatistics, School of Public Health, Addis Ababa University, Addis Ababa, Ethiopia; ^3^Department of Epidemiology and Biostatistics, Addis Continental Institute of Public Health, Addis Ababa, Ethiopia; ^4^Department of Reproductive Health and Population, Addis Continental Institute of Public Health, Addis Ababa, Ethiopia

## Abstract

**Background:**

Access to and utilization of facility delivery services is low in Ethiopia. The government of Ethiopia introduced a free delivery service policy in all public health facilities in 2013 to encourage mothers to deliver in health facilities. Examining the effect of this intervention on the utilization of delivery services is very important.

**Objective:**

In this study, we assessed the effect of provisions of free maternity care services on facility-based delivery service utilization in central Ethiopia.

**Methods:**

Data on 108 time points were collected on facility-based delivery service utilization (72 pre- and 36 postintervention) for a period of nine years from July 2007 to June 2016. Routine monthly data were extracted from the District Health Information System and verified using data from the delivery ward logbooks across the study facilities. An interrupted time-series analysis was conducted to assess the effect of the intervention.

**Results:**

The implementation of the free delivery services policy has significantly increased facility deliveries. During the study period, there was a statistically significant increase in the number of facility-based deliveries after the 24^th^ and 36^th^ months of intervention (*p* < 0.05). Program effects on the use of public facilities for deliveries were persisted over a longer exposure period.

**Conclusion:**

The findings suggested that the provision of free delivery services at public health facilities increased facility delivery use. The improved utilization of facility delivery services was more marked over a longer exposure period. Policy-makers may consider mobilizing the communities aware of the program at its instigation.

## 1. Introduction

Improving maternal service utilization to attain sustainable development goals and to reduce the noticeably high maternal and neonatal mortality in low-income countries remains a pressing priority [1]. Maternal mortality is generally high in Sub-Saharan African (SSA) countries accounting for 66% of maternal deaths globally [1]. Ethiopia is one of the countries with high maternal mortality, 412 per 100,000 live births [2]. Efforts to end preventable maternal and neonatal deaths consider universal coverage of facility delivery services as one of the key interventions [1, 3].

Facility-based delivery is vital in the reduction of maternal mortality; attaining high coverage alone can prevent up to 54% of maternal deaths [4]. Yet only 46% of women in SSA gave birth in health facilities [5]. Thus, to increase the utilization of facility delivery, many African countries have initiated free maternity services (FMS) [6, 7]. Studies across many SSA countries generally suggest increased utilization of facility delivery with the removal of user fees [5, 8, 9].

A study by McKinnon et al. for a number of SSA countries confirms that the removal of user fees is associated with an increase of 3.1 facility-based deliveries per 100 live births [7].

In Zambia, following the removal of user fees, facility-based deliveries did not increase significantly for public facilities, but a significant positive effect for private (faith-based) facilities with limitations because it only accounts for five percent of the sample [10]. A study in Ghana showed variation in coverage of delivery fees between districts [11].

In Kenya, recent evidences related to facility-based deliveries are heterogeneous. One article mentions a significant increase in the number of deliveries over a longer exposure period after abolition [12], while another reports that the increase in the number of deliveries was observed during the early phase of the program [13]. The instance of unsustained increase in facility delivery service utilization following the removal of user fees was also reported in Nepal's study [14, 15].

However, only a few studies have used rigorous analysis adjusting for underlying trends so far [6, 7, 16, 17]. Hence, evidence on the effect of free delivery service policy on utilization facility delivery remains limited and mixed.

In Ethiopia, despite the implementation of the free delivery services policy in the country since 2013 [18], to date, 26% of mothers delivered in health facilities [2]. Since the initiation of the free maternal care services in Ethiopia, no study has been undertaken to assess its effect on the utilization of health facilities for delivery services. Thus, this study was conducted to assess the effect of the provisions of FMS on the utilization of facility-based delivery services in the East-Shewa Zone, Central Ethiopia.

## 2. Methods

### 2.1. Study Setting

The study was conducted in the East-Shewa zone, one of the sixty-eight zones in Ethiopia. The Zone was selected because it was one of the administrative zones that began implementing the national free delivery service intervention at the initiation phase in 2013 in all public health centres [18]. The Zone had an estimated population of 1,967,077 inhabitants based on the projected population of Ethiopia, 2017 [19].

The Ethiopian health care system encourages normal deliveries to be conducted at health centers, and the need to refer only complicated cases to hospitals [18].

The government has created a primary level care consisting of health posts (HPs), health centers (HCs), and primary/district hospitals which are all connected to each other through a referral system [18]. A free ambulance service is organized in every district to facilitate referral for obstetrics emergencies [20].

### 2.2. Study Design

The study utilized an interrupted time series design, which is appropriate to compare changes in the utilization of facility delivery services before and after the introduction of the free service intervention [21–23]. Given that there was a definite point in time when the implementation of the policy began and data were available in the health facilities for the time period before and after the intervention, interrupted time-series design is the ideal method.

### 2.3. Study Facilities and Sampling Strategy

The process of selecting health facilities had two phases: we first considered all of the 25 HCs within the zone. Then, we made a preliminary assessment and initially identified 13 HCs within the zone that had complete facility birth data 2007-2016; the rest 11 had fragmentary delivery ward logbooks in place, and the data were confused with omitted months, year, torn page, and multiple outliers and one HC (Methehara) had only 3 years data 2014-2016 because of flood in 2013. Hence, the 12 HCs were excluded from the sampling frame.

**Figure 1:**
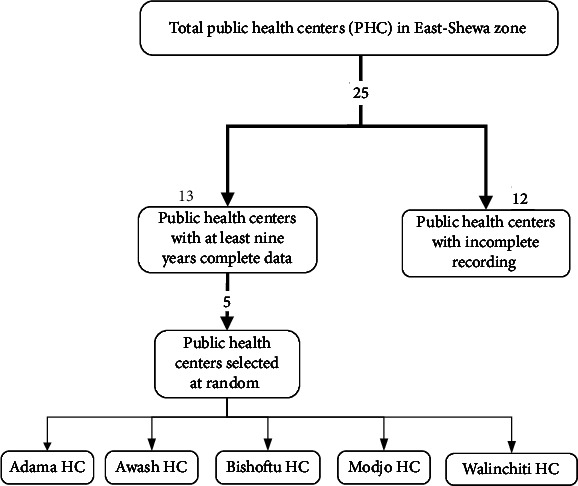
A flow diagram showing the steps taken for selecting the facilities within the East-Shewa zone.

In the second phase, of the 13 HCs which had the last nine years' complete data, 5 HCs were randomly selected. A flow diagram showing the steps taken for selecting facilities is presented in Figure 1.

### 2.4. Data Collection

The outcome variable was the total number of deliveries taking place in public health facilities per month. Information on the intervention period (both before and after the launch of the policy on July 1, 2013) was obtained from the East-Shewa health office.

Data were extracted from district health records using a structured data retrieving form. These were then verified using facility-based delivery ward logbooks to ensure data accuracy in the study. The data were collected for the period between July 2007 and June 2016, a total of 108 months, 72 pre- and 36 postintervention months. Experienced record officers were recruited, trained, and used in the data collection. The first officer collected the data from each study site. The second officer counter checked and verified whether the collected data were similar to what was in the study facilities' logbooks. Only verified data were used for analysis.

### 2.5. Data Analysis

The data were verified and cleaned using an Excel spreadsheet then exported to Stata version 12 for analysis. We carried out an interrupted time series (ITS) analysis (segmented regression model) to estimate the effect of the policy on facility deliveries [17] (clean data found as supplementary file 1). The segmented regression model used for the study gives three coefficient *β*1, *β*2, and *β*3:

(1) $$\begin{array}{c} Y_{t}=\beta_{0}+\beta_{1}T_{t}+\beta_{2}X_{t}+\beta_{3}X_{t}T_{t}+\varepsilon_{t}, \end{array}$$

where *Y*_*t*_ is the number of facility-based deliveries (FBD), *β*0 estimates the baseline level FBD at the beginning of the period, *β*1 estimates the change in the number of FBD until the free delivery service policy, *β*2 is the change in FBD immediately after the free delivery service policy, and *β*3 is the difference in the trends of FBD between pre- and postchanges in policy (effect of the intervention over time).

The covariates are defined as follows: *T*_*t*_ is the time from the start to the end of the observation period and *X*_*t*_ is a dummy variable coded 0 and 1 for periods preintervention (from July 2007 to June 2013) and postintervention (July 2013 to June 2016), respectively; *X*_*t*_*T*_*t*_ is an interaction term between the time and intervention dummy; and *ε*_*t*_ is the error term.

Estimates for regression coefficients corresponding to two standardized effect sizes are obtained: a change in level (step change, i.e., *β*2) and a change in trend before the intervention (*β*1). The change in trend after the intervention (*β*3) is the sum of the preintervention slope and the change in level. Slope and level regression coefficients were important in calculating the 95% confidence intervals. The *p* values demonstrated the significance of the effect of the free delivery service policy. Percentages were calculated to estimate the relative effect of the policy.

Policy effect equates = FBD with policy − FBD without policy, where FBD without policy = *β*0 (constant) + *β*1T_t_ and FBD with policy = *β*2 × *X*_*t*_ + *β*3 × *X*_*t*_*T*_*t*_.

We estimated the absolute effect of the free facility-based delivery policy at 12, 24, and 36 months or at the 1^st^, 2^nd^, and 3^rd^ year of implementation, respectively, with the derivative of the policy shown here FBD = *β*2 × *X*_*t*_ + *β*3 × *X*_*t*_*T*_*t*_.

The main aim of the regression analysis here is to measure the outcome of interest by keeping out the influence of potential time-varying confounders on the effect of the free delivery service intervention [22–24].

The analysis was done to determine the twelfth-month level effect after the policy execution date to monitor any short-term effects of the policy. The analysis for the twenty-fourth month was to demonstrate any midterm effects, whereas the analyses done at the thirty-sixth-month postintervention were to assess whether the policy had a long-term effect. Statistical significance was set at *p* ≤ 0.05.

## 3. Results

Using the data at the district level, we compiled zonal data for the 5 districts for a period of 9 years, 2007–2016. The list of omitted facilities is presented as supplementary file 1.

The population of women of reproductive age in the districts pre-post ranged from 7,119 vs. 8,696 at Modjo district to nearly eight times that in Adama, and the expected pregnancies ranged from 330 to 2,556 pregnancies per year across districts in the zone.

Table 1 depicts the population of women of reproductive age and service data before and after the free delivery care policy implementation. There was an increase in facility deliveries within the zone and across facilities except for the Adama health center.

**Figure 2:**
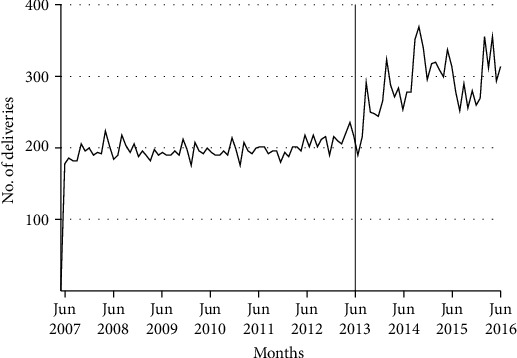
Trends for facility-based deliveries before and after implementation of the free maternity services policy in East Shewa zone (the line at 72 months).

Figure 2 shows the total number of monthly facility deliveries in the study health facilities. There was an immediate increase in the number of facility-based deliveries just following free delivery services policy implementation and then a temporary drop. The observed effect was smoothed by ITS analysis that involves a before-after comparison within a single population by creating control variables to estimate the counterfactual results, rather than a comparison with a control group. A trend was established for the before and after implementation of the intervention for facility-based deliveries with a goal of determining whether the intervention had a significantly greater effect than any underlying secular or seasonal trends. Throughout the postintervention period, the observed number of FBD under the free delivery services policy introduction was consistently higher than the expected number under the counterfactual results of nonintroduction of the free delivery services.

Table 2 shows the level and trend effects and the relative effects of the policy by year after the intervention. There had been a gradual increase of 0.8 facility-based deliveries per month before the implementation of free service (95% CI: 0.213–1.4; *p* < 0.01). Following the implementation of the free delivery services policy, there was an immediate increase of 320 facility-based deliveries conducted within health institutions (*p* < 0.05); 44% of this increase is directly attributable to the policy. There was a long-term effect of the policy by 350 (51% of which was due to the policy) more deliveries occurred in the third year (*p* < 0.001).

Thus, the average number of facility deliveries increased to 77, 98, and 118 per month in all facilities; in other words, 36, 44, and 51 of any 100 births in the health facilities could be attributed to the free delivery service implementation, compared with what it would have been without the free delivery service. These showed that the average number of facility deliveries increased by 36%, 44%, and 51% following the intervention at the 1^st^, 2^nd^, and 3^rd^ year of implementation, respectively. However, the increment in the 1^st^ year of the implementation was not statistically significant (Table 2).

## 4. Discussion

The implementation of the free delivery service policy in public health facilities in the study zone in 2013 was associated with a statistically significant increase in the utilization of facility-based deliveries. This finding is consistent with the results of the implementation of free maternal health care policies across several African countries [5, 7–9, 16]. A statistically significant increase in the use of public facilities for deliveries remained consistently high over the 2 years of exposure to the program. The effect size was also higher in longer phases. The high level of utilization of free delivery services over a long period of time in this study creates an opportunity to address the observed maternal and neonatal death toll.

This finding is in contrast with other studies, in which increased utilization of free delivery care services was documented during the early phase of the program implementation [6, 18].

Unlike the longer phase, the result for the likelihood of delivery in a public facility was statistically insignificant in the early phase, even though positive in sign in this study.

One of the reasons for observation of an insignificant effect in the early phase of the program might be low levels of awareness in the larger population about the availability of free delivery services. For example, in Ghana, communities apparently did not properly understand the abolition measure, despite various types of publicity effort [25]. Further, a qualitative finding conducted in three districts of Kenya supports these ideas, noting multiple challenges in program implementation, including inadequate stakeholder engagement and confusion on eligibility criteria and lack of reimbursement to health facilities for providing free delivery care.

It is possible that the free delivery services policy, despite being announced, was not completely internalized by either the health professionals or expectant mothers. Low levels of awareness in the larger population in the early phase may also explain the findings over the longer exposure period. The findings suggest the need to mobilizing communities (occasioned by women's group called “Women's Health Development Army (WHDA)”) [26–28] to be aware of the availability of free delivery and a 24-hour ambulance services for laboring women [29].

There have been improvements in the utilization of maternal health services as expected; studies have shown that facility deliveries usually increase following similar fee exemption policies [10, 11, 15, 16].

Although the rise in facility-based deliveries within the study zone is promising, considerable work remains to make the communities initially aware of the availability of free maternal services and to demand utilization. Studies suggest many possible explanations that could range from low coverage of the free delivery services due to overflow of users vs. lack/delays of reimbursement or that the policy might not evenly be implemented across treated districts [9, 30, 31].

Numerous studies showed that user charges are not the only financial determinants of seeking delivery services—unavailability and high costs of transport services and other delivery-related service costs are also financial barriers to access delivery services [32–34].

Further, variations in the attribute of free delivery service results are expected, since user fees are not the only factors to determine the use of facilities for delivery, and therefore, the relative importance of the fees compared to other determinants is likely to vary from time to time. Thus, the potential impact and magnitude of the policy change are likely to also vary accordingly [9].

The strengths of the current study include that data were extracted over a long period of time to absorb the effect of yearly fluctuations, the quality of data obtained from district records was verified by reviewing the delivery ward logbooks by trained and expert data collectors, and the assumptions for interrupted time series analysis were fulfilled. These are clearly differentiated time periods and sequential measures with a clear outcome of interest [22].

While the use of an interrupted time series (ITS) design is a strength of the study, it also presents a limitation, in that it is not possible to identify contextual factors associated with differences in the magnitude of service utilization.

The free maternal health care policy has been implemented in all public health facilities. This brought limitations in getting control groups (public health facilities where the policy was not implemented) for comparisons. Although most of the findings from this study are consistent with other studies, externalization of findings to depict the national picture in the implementation of the free maternal health care policy may be questioned given the shortcomings to address other contextual factors affecting health facility delivery services utilization in this study.

Further, it is possible that the data abstracted manually by the record officer are susceptible to reporting bias. This risk may be smoothed by the fact that data were verified using health facility records.

The fact that this study demonstrated free maternity services can significantly increase the number of facility delivery shows cost is one of the major barriers to facility-based delivery service utilization; however, it may not be the only factor contributing to the utilization of health facility for delivery as the majority of mothers in the study areas still deliver at home. Therefore, there is a need to consider other determinants that might be very crucial for the creation of delivery service demand by mothers. To comprehensively understand the effect of the free delivery policy, future qualitative studies should be carried out to understand other determinants in the study area with the context of the free delivery care policy.

Consistency in the direction of effects suggests robust evidence that user fees have an effect on the utilization of health facility delivery services in this study.

## 5. Conclusion

The findings suggest that the provision of free delivery services at public health facilities increased facility delivery use. The improved utilization of facility delivery services was more marked over a longer exposure period. The high level of utilization of free delivery services over a long period of time in this study creates an opportunity to address the observed maternal and neonatal death toll.

This finding implies that removal of user fees for facility delivery service utilization over a long period of exposure may serve as an important public health measure to address the observed low level of facility delivery and thereby reduce the highest maternal and neonatal death toll.

Thus, policy-makers may consider mobilizing communities to be aware of the program at its instigation.

## Figures and Tables

**Table 1:** The population of women of reproductive age and service data before and after the free delivery care policy implementation.

	Preintervention	Postintervention
Population data	July 2007-June 2013	July 2013-June2016
Zonal women of reproductive age	2,169,190	1,261,067
Expected deliveries	82,429	47,920
Average expected deliveries per month	1,145	1,331
Total facility-based deliveries (FBD) (ratio)	14,270 (17.3%)	10,460 (21.83%)
Average facility deliveries per month	198	291
FBD by health facilities		
Adama health center	6,686	3,138
Awashmelkasa health center	850	1,905
Bishoftu health center	863	1,488
Modjo health center	3,991	1,761
Walinchiti health center	1,880	2,167

**Table 2:** Parameter estimates, standard errors, and *p* values of a segmented regression model predicting the average number of deliveries per month in East-Shewa zone, central Ethiopia, 2007-2016.

Independent variables	Coefficient	Standard error	*p* value	RE%
Model (correcting for the first-order autocorrelation)				
Initial level of facility deliveries (*β*_0_)	173.867	8.791	0.000^∗∗∗^	
Preintervention slope (*β*_1_)	0.807	0.299	0.008^∗∗^	
Immediate intervention effect (*β*_2_)	56.793	10.934	0.000^∗∗∗^	
Postintervention slope (*β*_3_)	1.698	16.817	0.009^∗∗^	
Level effect				
1^st^ year	290	16.542	0.452	36.2
2^nd^ year	320	11.828	0.015^∗^	44
3^rd^ year	350	10.934	0.000^∗∗∗^	51

Note: ^∗∗∗^*p* < 0.001, ^∗∗^*p* < 0.01, ^∗^*p* < 0.05.

## Data Availability

The data supporting the conclusions drawn in this study have been included in the article. However, the datasets underlying the findings of our study are contained within the supplementary materials, as supplementary file 2.

## Abbreviations

ANC: Antenatal care
BEOC: Basic emergency obstetrics care
HC: Health center
HPs: Health posts
MCH: Maternal and child health
MDG: Millennium development goals
PHCU: Primary health care unit
SSA: Sub-Saharan Africa
SDGs: Sustainable development goals
WHDA: Women's health development army.

## Supplementary Materials

Supplementary Materials Supplementary file 1: Table showing the list of health centers that removed user fees in July 2013 and those marked “X” were incomplete and discarded. Supplementary file 2: Table showing a clean dataset used for the analysis of facility delivery in East-Shewa zone, 2007-2016.

## References

[1] United Nations (2015). *Sustainable Development Goals*.

[2] Central Statistical Agency (CSA) [Ethiopia] (2016). *Ethiopia Demographic and Health Survey 2016: Key Indicators Report*.

[3] Campbell O. M. R., Graham W. J. (2006). Strategies for reducing maternal mortality: getting on with what works. *The Lancet*.

[4] Bhutta Z. A., Das J. K., Bahl R. (2014). Can available interventions end preventable deaths in mothers, newborn babies, and stillbirths, and at what cost?. *The Lancet*.

[5] Langlois É. V., Karp I., Serme J. D. D., Bicaba A. (2016). Effect of a policy to reduce user fees on the rate of skilled birth attendance across socioeconomic strata in Burkina Faso. *Health Policy and Planning*.

[6] Dzakpasu S., Powell-Jackson T. (2014). Impact of user fees on maternal health service utilization and related health outcomes: a systematic review. *Health Policy and Planning*.

[7] McKinnon B., Harper S., Kaufman J. S., Bergevin Y. (2015). Removing user fees for facility-based delivery services: a difference-in-differences evaluation from ten sub-Saharan African countries. *Health Policy and Planning*.

[8] Manthalu G., Yi D., Farrar S., Nkhoma D. (2016). The effect of user fee exemption on the utilization of maternal health care at mission health facilities in Malawi. *Health policy and planning*.

[9] Leone T., Cetorelli V., Neal S., Matthews Z. (2016). Financial accessibility and user fee reforms for maternal healthcare in five sub-Saharan countries: a quasi-experimental analysis. *BMJ Open*.

[10] Chama Chiliba C. M., Koch S. F. (2016). An assessment of the effect of user fee policy reform on facility based deliveries in rural Zambia. *BMC Research Notes*.

[11] Johnson F. A. (2016). A geospatial analysis of the impacts of maternity care fee payment policies on the uptake of skilled birth care in Ghana. *BMC Pregnancy and Childbirth*.

[12] Gitobu C. M., Gichang P. B., Mwanda W. O. (2018). The effect of Kenya’s free maternal health care policy on the utilization of health facility delivery services and maternal and neonatal mortality in public health facilities. *BMC Pregnancy and Childbirth*.

[13] Tama E., Molyneux S., Waweru E., Tsofa B., Chuma J., Barasa E. (2018). Examining the implementation of the free maternity services policy in Kenya: a mixed methods process evaluation. *International Journal of Health Policy and Management*.

[14] Witter S., Khadka S., Nath H., Tiwari S. (2011). The national free delivery policy in Nepal: early evidence of its effects on health facilities. *Health policy and planning*.

[15] Lamichhane P., Sharma A., Mahal A. (2017). Impact evaluation of free delivery care on maternal health service utilisation and neonatal health in Nepal. *Health Policy and Planning*.

[16] Dzakpasu S., Soremekun S., Manu A. (2012). Impact of free delivery care on health facility delivery and insurance coverage in Ghana’s Brong Ahafo region. *PLoS One*.

[17] Wagner A. K., Soumerai S. B., Zhang F., Ross‐Degnan D. (2002). Segmented regression analysis of interrupted time series studies in medication use research. *Journal of Clinical Pharmacy and Therapeutics*.

[18] FMoH (2010). *health sector development program IV 2010-15*.

[19] Central Statistical Agency (CSA) [Ethiopia] (2013). *Population projection of Ethiopia for all regions at Wereda level from 2014 – 2017*.

[20] Bayu H., Adefris M., Amano A., Abuhay M. (2015). Pregnant women’s preference and factors associated with institutional delivery service utilization in Debra Markos town, north West Ethiopia: a community based follow up study. *BMC Pregnancy and Childbirth*.

[21] Taljaard M., McKenzie J. E., Ramsay C. (2014). The use of segmented regression in analysing interrupted time series studies: an example in pre-hospital ambulance care. *Implementation Science*.

[22] Kontopantelis E., Doran T., Springate D. A., Buchan I., Reeves D. (2015). Regression based quasi-experimental approach when randomisation is not an option: interrupted time series analysis. *BMJ Open*.

[23] Bernal J. L., Cummins S., Gasparrini A. (2016). Interrupted time series regression for the evaluation of public health interventions: a tutorial. *International Journal of Epidemiology*.

[24] Thamer M., Zhang Y., Lai D., Kshirsagar O., Cotter D. (2013). Influence of safety warnings on ESA prescribing among dialysis patients using an interrupted time series. *BMC Nephrology*.

[25] Witter S., Arhinful D. K., Kusi A., Zakariah-Akoto S. (2007). The experience of Ghana in implementing a user fee exemption policy to provide free delivery care. *Reproductive Health Matters*.

[26] Chankham T., Yamamoto E., Reyer J. A. (2017). Knowledge of free delivery policy among women who delivered at health facilities in Oudomxay Province, Lao PDR. *Nagoya journal of medical science*.

[27] Girmaye M., Berhan Y. (2016). Skilled antenatal care service utilization and its association with the characteristics of women’s health development team in Yeky District, South-West Ethiopia: a multilevel analysis. *Ethiopian Journal of Health Sciences*.

[28] Nahar S., Banu M., Nasreen H. E. (2011). Women-focused development intervention reduces delays in accessing emergency obstetric care in urban slums in Bangladesh: a cross-sectional study. *BMC Pregnancy and Childbirth*.

[29] Prinja S., Jeet G., Kaur M., Aggarwal A. K., Manchanda N., Kumar R. (2014). Impact of referral transport system on institutional deliveries in Haryana, India. *The Indian Journal of Medical Research*.

[30] Ajayi A. I. (2017). Who benefits from free institutional delivery? Evidence from a cross sectional survey of North Central and Southwestern Nigeria. *BMC Health Services Research*.

[31] De Allegri M., Ridde V., Louis V. R. (2011). Determinants of utilisation of maternal care services after the reduction of user fees: a case study from rural Burkina Faso. *Health Policy and Planning*.

[32] Kruk M. E., Mbaruku G., Rockers P. C., Galea S. (2008). User fee exemptions are not enough: out-of-pocket payments for 'free' delivery services in rural Tanzania. *Tropical Medicine & International Health*.

[33] Hatt L. E., Makinen M., Madhavan S., Conlon C. M. (2013). Effects of user fee exemptions on the provision and use of maternal health services: a review of literature. *Journal of health, population, and nutrition*.

[34] Masiye F., Kaonga O. (2016). Determinants of healthcare utilisation and out-of-pocket payments in the context of free public primary healthcare in Zambia. *International Journal of Health Policy and Management*.

