# Supplementary material for: Effect of Implementing a Free Delivery Service Policy on Women's Utilization of Facility-Based Delivery in Central Ethiopia: An Interrupted Time Series Analysis
Source: J Pregnancy. 2020 Dec 12;2020:8649598. doi: 10.1155/2020/8649598 (PMC7752279; doi:10.1155/2020/8649598)
Supplement: Supplementary Materials — Supplementary file 1: Table showing the list of health centers that removed user fees in July 2013 and those marked “X” were incomplete and discarded. Supplementary file 2: Table showing a clean dataset used for the analysis of facility delivery in East-Shewa zone, 2007-2016. [file 8649598.f1.zip › Supplementary files/Supplementary file 1.doc]

| Supplementary file 1: Table showing the list of health Centers that removed user fees in July 2013 and those marked ‘X’ were incomplete and discarded | | | |
| --- | --- | --- | --- |
| Name of HC | location | Status of record keeping | |
|  |  | Adequate | Inadequate |
| 1. Modjo HC | Madjo | ✓ |  |
| 2. Meki HC | Meki | ✓ |  |
| 3. Batu 1 HC | Zeway | ✓ |  |
| 4. Batu 2 HC | Zeway | ✓ |  |
| 5. Walinchity HC | Walenchiti | ✓ |  |
| 6. Bishoftu HC | Bishoftu | ✓ |  |
| 7. Adama HC | Adama | ✓ |  |
| 8. Geda HC | Adama | ✓ |  |
| 9. Awashmelkasa HC | Awash melkasa | ✓ |  |
| 10. Bulbula HC | Near to zeway | ✓ |  |
| 11. Algae HC | Around Zeway | ✓ |  |
| 12. Shoa Alemtena HC | Around Wanji | ✓ |  |
| 13. Adulala HC | 10km away beshftu | ✓ |  |
| 14. Metahara HC | Metahara |  | × |
| 15. Adami Tulu HC | Adamitulu |  | × |
| 16. Bote HC | Adamitulu |  | × |
| 17. Chafedonsa HC | Around beshuftu |  | × |
| 18. Dire HC | Around Meki |  | × |
| 19. Doni HC | Around Walenchiti |  | × |
| 20. Ejere HC | Around Modjo |  | × |
| 21. Bilitti HC | Around Meki |  | × |
| 22. Dobbi HC | Around Meki |  | × |
| 23. Godino HC | Around beshuftu |  | × |
| 24. Jido Kombolcha HC | Around Zeway |  | × |
| 25. Dongorota HC | Around beshuftu |  | × |
